# Crystal Structures of the ATPase Domains of Four Human Hsp70 Isoforms: HSPA1L/Hsp70-hom, HSPA2/Hsp70-2, HSPA6/Hsp70B', and HSPA5/BiP/GRP78

**DOI:** 10.1371/journal.pone.0008625

**Published:** 2010-01-11

**Authors:** Magdalena Wisniewska, Tobias Karlberg, Lari Lehtiö, Ida Johansson, Tetyana Kotenyova, Martin Moche, Herwig Schüler

**Affiliations:** Structural Genomics Consortium, Karolinska Institutet, Stockholm, Sweden; University of Cambridge, United Kingdom

## Abstract

The 70-kDa heat shock proteins (Hsp70) are chaperones with central roles in processes that involve polypeptide remodeling events. Hsp70 proteins consist of two major functional domains: an N-terminal nucleotide binding domain (NBD) with ATPase activity, and a C-terminal substrate binding domain (SBD). We present the first crystal structures of four human Hsp70 isoforms, those of the NBDs of HSPA1L, HSPA2, HSPA5 and HSPA6. As previously with Hsp70 family members, all four proteins crystallized in a closed cleft conformation, although a slight cleft opening through rotation of subdomain IIB was observed for the HSPA5-ADP complex. The structures presented here support the view that the NBDs of human Hsp70 function by conserved mechanisms and contribute little to isoform specificity, which instead is brought about by the SBDs and by accessory proteins.

**Enhanced version:**

**This article can also be viewed as an enhanced version in which the text of the article is integrated with interactive 3D representations and animated transitions. Please note that a web plugin is required to access this enhanced functionality. Instructions for the installation and use of the web plugin are available in Text S1.**

## Introduction

The heat shock-70 proteins (Hsp70s) are chaperones involved in crucial cellular functions in all kingdoms of life. Hsp70-family function, structure, and mechanisms have been studied to great detail using cognate HSPA8/Hsc70 [1], stress-induced HSPA1/Hsp70-1/Hsp72 [2], [3] the ER-resident HSPA5/Bip [4], and the bacterial ortholog, DnaK [5]-[7]. Hsp70 proteins bind and release client polypeptides in a cycle that is coupled to an ATPase activity [8]. The overall domain structure is conserved: The N-terminal nucleotide binding domain (NBD) with ATPase activity is joined by a flexible linker to the C-terminal peptide substrate binding domain (SBD). Alternation between the ATP state with low affinity and high exchange rates for clients and the ADP state with high affinity and low client exchange rates is tightly regulated by several classes of associated proteins, or cochaperones [9]. Allosteric communication with the SBD and interactions with cochaperones and nucleotide exchange factors all critically depend on the conformation of the NBD. Therefore, understanding the conformational changes in the NBDs of Hsp70 proteins is key to understanding how the ATPase motor drives the client binding and release cycle of the Hsp70 machine.

The human genome contains over 40 hsp70 sequences. Many of them are pseudogenes, but at least eleven distinct genes located on several chromosomes are translated into protein [9]–[11]. These Hsp70 isoforms differ from each other by amino acid sequence, expression levels, subcellular localization, and induction in response to different cues. Both constitutively expressed and stress-induced Hsp70 proteins have been identified [12]. Constitutively expressed Hsp70 chaperones have housekeeping functions such as the folding of nascent polypeptides, protein translocation between cellular compartments, degradation of unstable and misfolded proteins, and regulation of assembly and disassembly of protein complexes. Other Hsp70 members can be induced by various cellular stresses, such as heat stress, heavy metals, radiation, ischemia, nitric oxide radicals, or other stimuli that activate stress transcription factors. Stress induced Hsp70s prevent accumulation of stress denatured proteins.

Several of the human cytosolic Hsp70 isoforms have yet to be extensively characterized. Heat shock 70-like protein-1 (HSPA1L, also called Hsp70-hom or Hsp70t) is a constitutively expressed, non-inducible cytosolic protein with high abundance in testis [13], [14]. Polymorphisms in the HSPA1L gene, predominantly in the region coding for the client binding domain, have been linked to disease [15], [16]. HSPA2/Hsp70-2 is a constitutively expressed protein with high levels in testis [17], [18]. HSPA2 is essential for maturation of male gametocytes [19] and is linked to male infertility [20], [21]. While it is undetectable in many tissues HSPA2 is involved in cancer cell survival [22]. The HSPA6/Hsp70B' gene is strictly inducible with no detectable basal expression [23], [24]. The HSPA6 protein is nuclear and cytosolic. HSPA6 induction is a sensitive biomarker of cellular stress; the protein appears transiently in response to heat stress whereas HSPA1A/Hsp72 levels persist for days [25].

The major Hsp70 protein of the endoplasmic reticulum is HSPA5, originally called immunoglobulin heavy chain binding protein (BiP), or glucose regulated protein-78 (GRP78) [26], [27]. HSPA5 has crucial roles in the assembly of ER proteins and protein complexes and the unfolded protein response [28]. Both HSPA5 and its interaction partners are linked to a number of diseases including infectious diseases, inherited diseases, and several types of cancer [29]–[31].

Despite the large number of NBD crystal structures determined under different nucleotide conditions, the conformational changes that ultimately drive the Hsp70 chaperone machine have never been visualized by X-ray crystallography of NBDs alone. Instead, the NBDs all crystallized in a closed conformation that is interpreted as resembling the ATP-bound state. Only crystal complexes of NBDs and nucleotide exchange factors have yielded structures with a partially opened nucleotide binding cleft [32]–[36]. Also, the small cleft between subdomains IA and IB that is expected to adopt different opening states in allosteric regulation of the SBD [7] looks similar in all NBD crystal structures. We used X-ray crystallography to address the question whether previously less well characterized human Hsp70 isoforms might adopt different conformations in the absence of binding partners. We determined the structures of the ATPase domains of HSPA1L, HSPA2, HSPA5, and HSPA6. Despite different nucleotides and divalent cations present during protein purification and crystallization these structures are highly similar to the crystal structure of HSPA1A and other previously determined Hsp70 NBDs. HSPA5 crystallized with ADP and calcium in the active site, and with calcium bound to a secondary site, whereas we observed the products of ATP hydrolysis (ADP and inorganic phosphate) and a divalent cation in the active sites of the remaining structures. We conclude that in isolation, the ATPase domains of these human Hsp70 isoforms have rather similar properties to those of the previously determined isoforms.

## Results and Discussion

**Figure 1:**
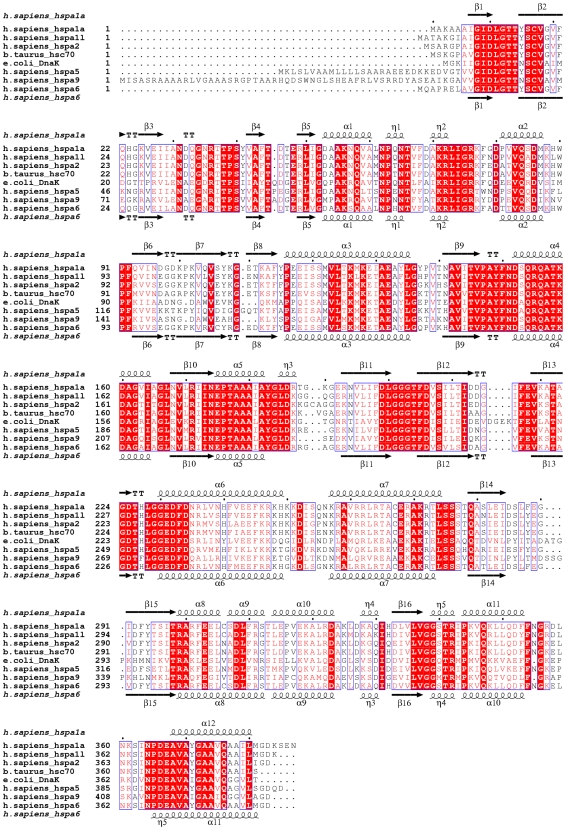
Relationship between the ATPase domains of human Hsp70 isoforms. Sequence alignment of the NBDs of selected Hsp70 proteins. Secondary structure elements are indicated for HSPA1A above and for HSPA6 below the alignment. Sequences shown are human HSPA1A (1HJO; gi:5123454); HSPA1L (3GDQ; gi:124256496); HSPA2 (3I33; gi:13676857); bovine Hsc70 (PDB entry 1YUW; gi:76253709), *E.coli* DnaK (1DKG; gi:16128008); HSPA5 (3IUC; gi:16507237); HSPA9 (no structure available; gi:24234688); and HSPA6 (3FE1; gi:34419635).

A common feature of Hsp70 ATPase domains is that, despite the large conformational changes they are predicted to undergo in their physiological context, their crystal structures are highly similar under different nucleotide conditions. We addressed the question whether this is also true for the NBDs of HSPA1L, HSPA2, HSPA5 and HSPA6. These share between 67 and 92% sequence identity with the NBD of the major stress inducible isoform, HSPA1A (Figure 1). We used a multiconstruct approach [37] to produce the NBDs of these human Hsp70 isoforms for structure determination by X-ray crystallography. We also produced the NBD of HSPA1A, the structure of which has been determined previously [38], [39]. All five proteins were straightforward to produce in *E. coli*, although the soluble expression levels for HSPA5/BiP were relatively low.

**Figure 2:**
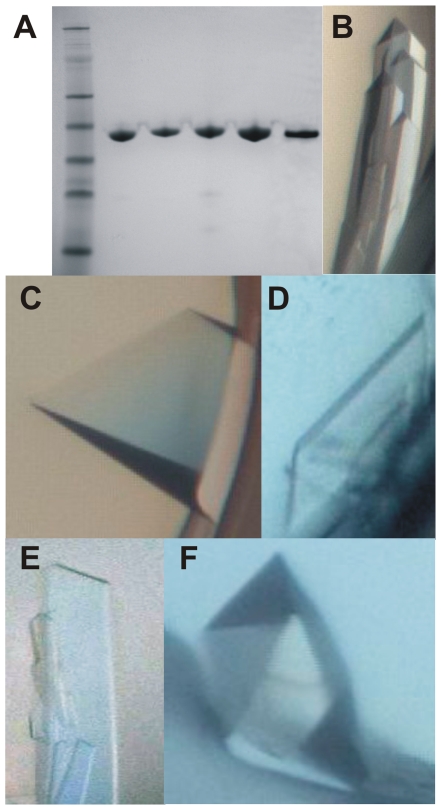
Purification and crystallization of Hsp70 isoforms. (A) Coomassie-stained SDS-polyacrylamide gel showing the purity of the crystallized proteins. (B-F) Examples of crystals grown under the conditions that yielded the datasets. (B) HSPA1A; (C) HSPA1L; (D) HSPA2; (E) HSPA5; (F) HSPA6.

**Figure 3:**
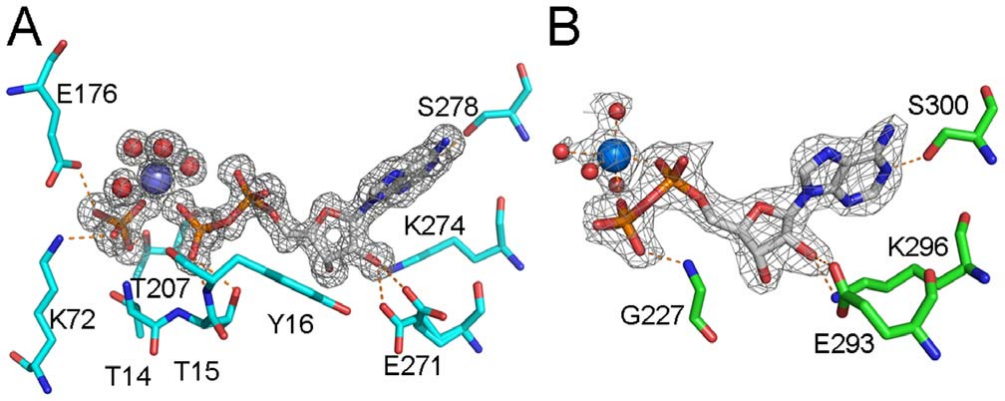
Electron density around the nucleotide in the NBDs of HSPA2 and HSPA5. Representative parts of the 2F_obs_-F_calc_ density maps around the nucleotide binding site (contoured at 1.5σ above the mean) of (A) HSPA2 with MgADP + P_i,_ and (B) HSPA5 with CaADP. Selected side chain and main chain atoms within hydrogen bonding distance are indicated.

Crystals of the five proteins were obtained with NBD constructs that contained either the full length N-terminus or short N-terminal truncations and with ADP and either Mg^2+^- or Mn^2+^-ions present in the crystallization solution (Figure 2 and Table 1). Crystals diffracted to a resolution of between 1.8 and 2.2 Å except for the HSPA2 crystals which diffracted to 1.3 Å. The structures were solved by molecular replacement (Table 2). In all ATPase domains except for that of HSPA5/BiP the products of ATP hydrolysis, including inorganic phosphate, were observed. Examples of the electron density around the bound nucleotide are shown for HSPA2 and HSPA5 (Figure 3).

**Table 1:** Summary of crystallization conditions.

	HSPA1A	HSPA1L	HSPA2	HSPA5	HSPA6
Salt	0.2M magnesium chloride	0.2M trimethyl amine n-oxide	0.2M ammonium acetate	0.2M calcium chloride	0.1M disodium hydrogen phosphate
Buffer	0.1M Bis-Tris	0.1M Tris	0.1M Bis-Tris	0.1M sodium acetate	0.1M citric acid
Precipitant	25% PEG-3350	26% PEG monomethyl ether-2000	25% PEG-3350	20% PEG-6000	16% PEG-300
pH	5.5	8.5	5.5	5	3.2
Additive	ADP, MgCl_2_	ADP, MnCl_2_	ADP, MgCl_2_	ADP, MgCl_2_	ADP, MgCl_2_
Temperature	4°C	4°C	4°C	20°C	20°C

**Table 2:** Summary of data acquisition and structure refinement details*.

Protein	HSPA1A	HSPA1L	HSPA2	HSPA5	HSPA6
PDB entry	3JXU	3GDQ	3I33	3IUC	3FE1
Search model	1BA0	1BA0	3FE1	3I33	2E88
Ligand	ADP, PO_4_, Mg^2+^	ADP, PO_4_, Mn^2+^, GOL	ADP, PO_4_, Mg^2+^, GOL	ADP, Ca^2+^	ADP, PO_4_, Mg^2+^, Cl, PGE
**Data collection**					
X-ray source	Cu-Kα radiation	DIAMOND I03	ESRF ID23-1	BESSY 14.1	BESSY 14.1
Wavelength (Å)	1.54166	0.98000	0.97930	0.91841	0.91841
Space group	P212121	P212121	P212121	P1	C121
Cell dimensions					
*a*, *b*, *c* (Å)	46.013, 63.298, 144.197	70.07, 70.7, 97.5	48.180, 78.6, 93.99	48.212, 51.795, 94.994	236.45, 105.4, 73.59
*α, β, γ* (°)	90, 90, 90	90, 90, 90	90, 90, 90	98.85, 94.88, 117.61	90, 101.04, 90
Resolution (Å)	26.43–2.14 (2.24–2.14)	25.0–1.8 (1.85–1.8)	26.26–1.3 (1.39–1.3)	35.0–2.13 (2.13–2.19)	35.0–2.2 (2.28–2.2)
R_merge_	0.093 (0.297)	0.07 (0.2)	0.136 (0.266)	0.13 (0.8)	0.12 (0.6)
I/σ(I)	20.6 (6.87)	27.9 (9.36)	11.6 (0.5)	11.42 (2.9)	10.54 (2.8)
Completeness (%)	98.3 (88.8)	99.3 (93.2)	99.2 (99.3)	94.4 (95.3)	98.6 (98.1)
Redundancy	12.7 (8.45)	13.7 (7.9)	6.9 (7.0)	3.8 (3.7)	3.9 (3.9)
Refinement					
Resolution (Å)	10.54–2.14	23.83–1.8	25.56–1.3	29.12–2.4	33.69–2.2
No. reflections	22346	42856	83300	28620	88609
R_work_ †/R_free_ ‡ (%)	20.9/26.4	17.2/20.2	18.4/19.9	19.4/27.0	20.1/23.8
Molecules/a.u.	1	1	1	2	3
No. atoms					
Protein	2930	2936	2934	5912	8868
Ligands	33	39	39	58	110
Water	202	309	345	399	333
*B*-factors (Å^2^)					
Protein	19.26	19.73	12.29	22.0	25.45
Ligands	11.89	12.84	8.2	12.34	19.78
Water	21.35	26.91	22.32	24.29	25.8
R.m.s deviations					
Bond lengths (Å)	0.013	0.012	0.007	0.016	0.013
Bond angles (°)	1.415	1.367	1.212	1.588	1.38
Ramachandran plot (%)					
Favored regions	98.1	99.2	99.1	98.4	98.5
Additionally allowed regions	1.9	0.8	0.9	1.6	1.5

* Values for the highest resolution shell are shown in parentheses.

† R_work_ is defined as Σ||F_obs_|−|F_calc_|| / Σ|F_obs_|, where F_obs_ and F_calc_ are observed and calculated structure-factor amplitudes, respectively.

‡ R_free_ is the R factor for the test set (5–10% of the data).

**Figure 4:**
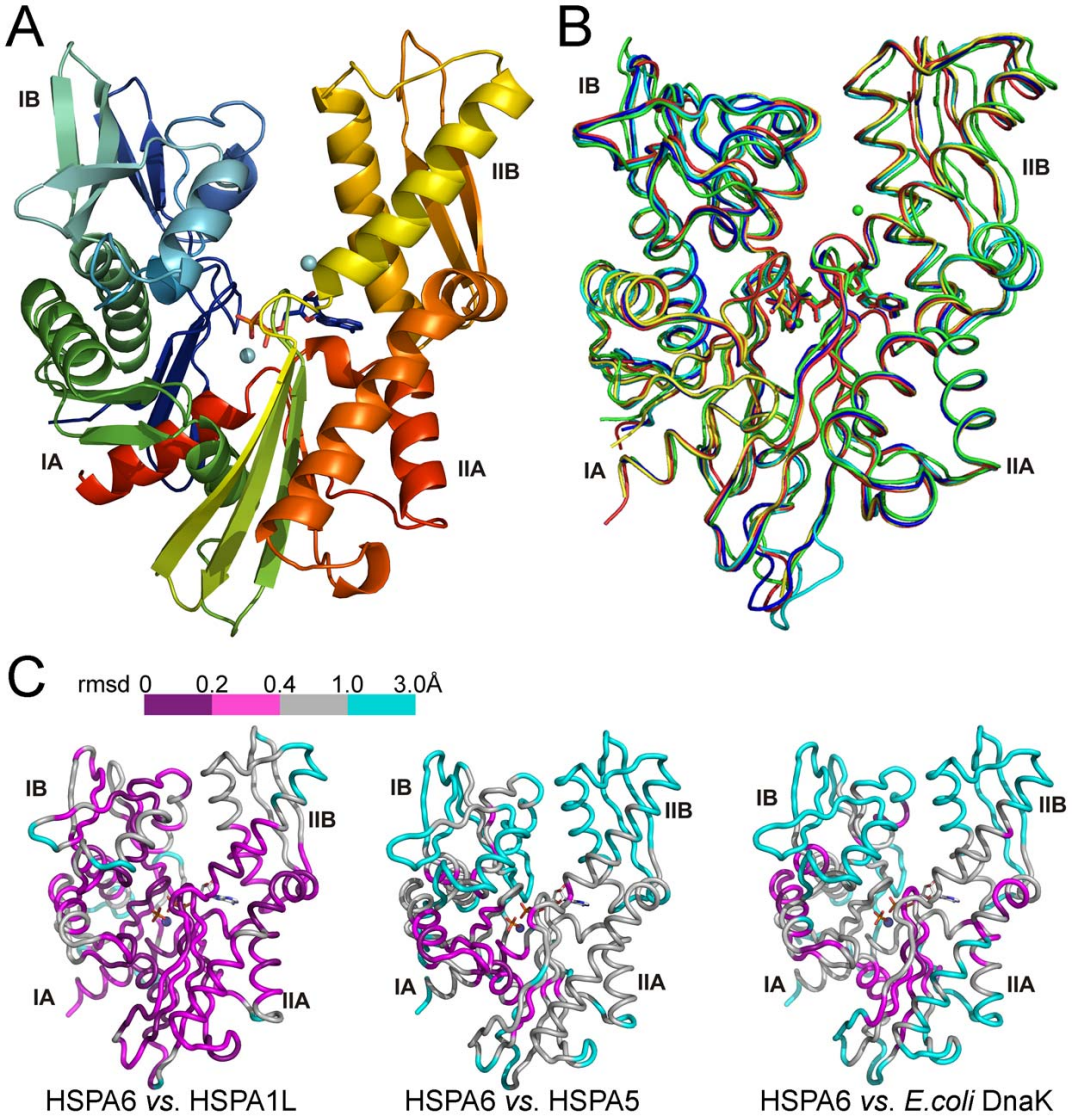
Crystal structures of the human Hsp70 ATPase domains. (A) The structure of the HSPA5/BiP NBD in complex with CaADP at 2.4 Å resolution. (B) Superposition of the five Hsp70 NBD structures determined in this study. Dark blue, HSPA1A; yellow, HSPA1L; cyan, HSPA2; red, HSPA6; green, HSPA5/BiP. (C) Cartoon representation of HSPA6, colored to illustrate rms differences in Cα-positions between HSPA6 and HSPA1L (left), HSPA6 and HSPA5 (center), and HSPA6 and DnaK (right).

The overall structures of the four human Hsp70 ATPase domains that were determined here for the first time all closely resemble the structure of HSPA1A and related previously determined structures (Figure 4). The HSPA1A NBD structure determined by us was virtually identical with the HSPA1A NBD structure published previously (PDB entry 1HJO; [39]). The canonical Hsp70 fold, with the common placement of secondary structural elements, was also observed for HSPA5/BiP, the least conserved member of the five proteins studied by us (Figure 4A). Pairwise comparison among these NBDs shows that for ∼70% of the backbone traces the rms difference is below 0.5 Å (Figure 4B). The largest rms deviation in the Cα positions was found for HSPA5, where the overall rms value is higher than 1.3 Å (only 17% of the backbone trace shows an rms difference lower than 0.5 Å). To illustrate this we color coded the rmsd in Cα atom positions between the pairs HSPA6 - HSPA1L, HSPA6 - HSPA5, and HSPA6 - *E.coli* DnaK, and mapped them onto the structure of HSPA6 (Figure 4C). This analysis shows that the most prominent difference between the HSPA5 and the canonical Hsp70 structures is a shift in subdomain IIB.

The slightly opened nucleotide binding cleft of ADP-bound HSPA5 distinguishes it from the set of NBDs that crystallized with ADP and inorganic phosphate in the active site. Cleft opening in HSPA5 is brought about by rotation of subdomain IIB by approximately 5.8°, as determined using the DynDom server [40]. This mechanism of cleft opening is similar to that determined by crystallography of Hsp70 proteins in complex with nucleotide exchange factors [32]–[35], but differs from that observed for actin [41].

All five NBDs crystallized in the presence of ADP and either MgCl_2_ or MnCl_2_ (Table 1). Inorganic phosphate was present only in the solution for HSPA6. Nevertheless, electron density for inorganic phosphate was observed in the active site of four of these proteins (Table 2), which thus had the products of ATP hydrolysis bound to them. We conclude that at least HSPA1A, HSPA1L, and HSPA2 bound ATP in the expression host, hydrolyzed their bound ATP, and subsequently during crystallization never released the products of ATP hydrolysis into solution. Only the HSPA5 structure contained ADP and metal ion but not inorganic phosphate. Metal ion coordination geometry and distances suggested that here the bound divalent cation was calcium, which was present in the mother liquor. Thus HSPA5 may have lost magnesium and inorganic phosphate after cation exchange for calcium during crystallization.

A second calcium ion was found at the base of HSPA5 α-helix-6, tethered by the H252 hydroxyl and the E256 and D257 carboxylates. This site has previously been identified in human HSPA1 (1S3X) [38]. Conservation of the side chains involved in Ca^2+^-interactions in human and many other Hsp70s suggests a universal binding site, but to our knowledge no other crystal structures of any Hsp70 protein contain Ca^2+^ at this site. Given the millimolar concentrations of calcium in the ER we expect that HSPA5 may be partially regulated at the second site found in our crystal structure, whether in relation to ATP turnover or other activities [31]. Interestingly, this second metal binding site is implicated in ATP synthesis by mutagenesis of HSPA1 [42]. ATP synthesis was found to be dependent on both calcium ions and transient Hsp70 phosphorylation [42]. Also, both DnaK [43] and HSPA5 [44] are transiently phosphorylated, in a Ca^2+^-dependent manner, on the threonine side chain corresponding to HSPA5 T229. We speculate that HSPA5 and possibly other family members might be able to retain the products of ATP hydrolysis for ATP regeneration at a site in close proximity to the active site. The second calcium site might position the ADP pyrophosphate tail for phosphoester bond formation, while phosphorylated T229 (or an alternative side chain in the vicinity, such as strictly conserved HSPA5^T251^/DnaK^T225^) might act as phosphate donor. This pathway might be employed in situations of high ATP turnover or low ATP concentrations to secure vital cellular functions.

## Materials and Methods

### Protein Expression and Purification

The cDNAs coding for the full-length human HSPA1A, HSPA1L, HSPA2, HSPA5, and HSPA6 were obtained from the Mammalian Gene Collection (accession codes BC002453, BC034483, BC001752, BC020235, and BC035665, respectively). The sequence coding for residues HSPA1A^M1-N387^, HSPA1L^M1-K386^, HSPA2^P6-D386^, HSPA5^D26-D410^, and HSPA6^E6-D385^ were subcloned into expression vector pNIC-Bsa4 by ligation-independent cloning. The resulting expression constructs contained a hexahistidine tag and a TEV-protease cleavage site (MHHHHHHSSGVDLGTENLYFQS) at the N-termini.

Recombinant expression and purification of the proteins is described in detail on our webpage (http://www.thesgc.org/structures/). Briefly, each expression construct was transformed into *E. coli* strain BL21(DE3)R3 pRARE (Novagen) and the cultures were grown in Terrific Broth supplemented with 8 g/l of glycerol at 37°C. At an absorbance at 600 nm between 1 and 2 the temperature was lowered to 18°C, recombinant protein production was induced by addition of 0.5 mM isopropyl-β-d-thiogalactopyranoside, and cell growth was continued for 18 h at 18°C. Cells were harvested by centrifugation and the cell pellets were resuspended in 1.5 volumes/wet cell weight of lysis buffer (100 mM HEPES, 500 mM NaCl, 10% glycerol, 10 mM imidazole, 0.5 mM TCEP, pH 8.0, and one tablet of Complete EDTA-free protease inhibitor (Roche Biosciences) per 50 ml cell suspension). Before lysis, 4 µl (1000 U) of Benzonase (Novagen) was added per 50 ml cell suspension, and lysis was achieved by sonication. Cell debris was removed by centrifugation and the soluble fractions were filtered through a syringe filter (0.45 µm pore size). Cleared lysates were passed over 1-ml HiTrap Chelating columns (GE Healthcare) pre-equilibrated with buffer 1 (30 mM Hepes, 500 mM NaCl, 10% glycerol, 10 mM imidazole, pH 7.5, 0.5 mM TCEP). The columns were washed sequentially with buffer 1 and buffer 1 supplemented with 25 mM imidazole. Bound protein was eluted with buffer 1 containing 500 mM imidazole, loaded onto 16/60 Superdex-200 HiLoad columns (GE Healthcare), and gel filtration was performed in buffer 2 (30 mM Hepes, 300 mM NaCl, 10% glycerol, pH 7.5, 0.5 mM TCEP). Fractions were pooled based on gel filtration profiles and purity, TCEP was added to 2 mM, and the proteins were concentrated to 24.8 mg/ml (HSPA1A), 16.0 mg/ml (HSPA1L), 14.0 mg/ml (HSPA2), 31.0 mg/ml (HSPA5A), and 25.0 mg/ml (HSPA6). Proteins were typically more than 90% pure judged by SDS-PAGE analysis (Figure 2A). Protein construct masses were verified by TOF-MS analysis (results not shown). Aliquots were flash-frozen and stored at -80°C.

### Crystallization, Data Collection, Structure Solution and Refinement

Crystallization methods and conditions are summarized in Table 1. Crystals appeared after 2–28 days. For data collection crystals were briefly dipped in cryo solution containing 40% PEG 300, 0.15M Na_2_HPO_4_, 0.1M citric acid, 0.2M NaCl (HSPA6) or mother liquor containing 15%–20% glycerol (HSPA1A, HSPA1LA, HSPA2, and HSPA5) and flash-frozen in liquid nitrogen.

A single wavelength dataset for HSPA1A was collected with Cu-Kα radiation (1.54166 Å) on an X8 PROTEUM system equipped with a four-circle Kappa goniostat and a PLATINUM-135 CCD (all from Bruker AXS, Delft, Netherlands). SAINT (Bruker AXS) and XPREP (Bruker AXS) were used to process the data. Further datasets were collected at synchrotron beamlines at BESSY (BL14-1), Berlin, Germany (HSPA5 and HSPA6), DIAMOND (I03), Oxfordshire, UK (HSPA1L) and ESRF (ID23-1), Grenoble, France (HSPA2). Data sets were indexed, scaled, and reduced using the programs XDS [45] and SCALA [46]. All structures were solved by molecular replacement using MOLREP [47]. The structures were refined with RefMac5 [48] and model building was done using Coot [49]. For further details on data processing and refinement statistics, see Table 2. Geometry of the models was analyzed with Molprobity [50]. Sequence alignments were obtained using ESPript [51].

### Data Deposition

The atomic coordinates and structure factors have been deposited with the Protein Data Bank, www.rcsb.org (PDB entry codes: 3FE1 [HSPA6]; 3GDQ [HSPA1L]; 3I33 [HSPA2]; 3IUC [HSPA5]; 3JXU [HSPA1A]).

## Supporting Information

Datapack S1 Standalone iSee datapack - contains the enhanced version of this article for use offline. This file can be opened using free software available for download at http://www.molsoft.com/icm_browser.html.
(ICB)

Text S1 Instructions for installation and use of the required web plugin (to access the online enhanced version of this article).
(0.75 MB PDF)
